# Comparison between ^68^Ga-bombesin (^68^Ga-BZH3) and the cRGD tetramer ^68^Ga-RGD_4 _studies in an experimental nude rat model with a neuroendocrine pancreatic tumor cell line

**DOI:** 10.1186/2191-219X-1-34

**Published:** 2011-12-13

**Authors:** Caixia Cheng, Leyun Pan, Antonia Dimitrakopoulou-Strauss, Martin Schäfer, Carmen Wängler, Björn Wängler, Uwe Haberkorn, Ludwig G Strauss

**Affiliations:** 1 Clinical Cooperation Unit Nuclear Medicine, German Cancer Research Center, Heidelberg, Germany; 2 Department of Radiopharmaceutical Chemistry, German Cancer Research Center, Heidelberg, Germany; 3 University Hospital Munich, Department of Nuclear Medicine, Ludwig Maximilians-University Munich, Munich, Germany

**Keywords:** ^68^Ga-bombesin, ^68^Ga-RGD tetramer, PET, kinetic modeling, neuroendocrine tumors

## Abstract

**Objectives:**

Receptor scintigraphy gains more interest for diagnosis and treatment of tumors, in particular for neuroendocrine tumors (NET). We used a pan-Bombesin analog, the peptide DOTA-PEG_2_-[D-tyr^6^, β-Ala^11^, Thi^13^, Nle^14^] BN(6-14) amide (BZH3). BZH3 binds to at least three receptor subtypes: the BB1 (Neuromedin B), BB2 (Gastrin-releasing peptide, GRP), and BB3. Imaging of ανβ3 integrin expression playing an important role in angiogenesis and metastasis was accomplished with a ^68^Ga-RGD tetramer. The purpose of this study was to investigate the kinetics and to compare both tracers in an experimental NET cell line.

**Methods:**

This study comprised nine nude rats inoculated with the pancreatic tumor cell line AR42J. Dynamic positron emission tomography (PET) scans using ^68^Ga-BZH3 and ^68^Ga-RGD tetramer were performed (^68^Ga-RGD tetramer: *n *= 4, ^68^Ga-BZH3: *n *= 5). Standardized uptake values (SUVs) were calculated, and a two-tissue compartmental learning-machine model (calculation of *K*1 - *k*4 vessel density (VB) and receptor binding potential (RBP)) as well as a non-compartmental model based on the fractal dimension was used for quantitative analysis of both tracers. Multivariate analysis was used to evaluate the kinetic data.

**Results:**

The PET kinetic parameters showed significant differences when individual parameters were compared between groups. Significant differences were found in FD, VB, *K*1, and RBP (*p *= 0.0275, 0.05, 0.05, and 0.0275 respectively). The 56- to 60-min SUV for ^68^Ga-BZH3, with a range of 0.86 to 1.29 (median, 1.19) was higher than the corresponding value for the ^68^Ga-RGD tetramer, with a range of 0.78 to 1.31 (median, 0.99). Furthermore, FD, VB, *K*1, and RBP for ^68^Ga-BZH3 were generally higher than the corresponding values for the ^68^Ga-RGD tetramer, whereas *k*3 was slightly higher for ^68^Ga-RGD tetramer.

**Conclusions:**

As a parameter that reflects receptor binding, the increase of *K*1 for ^68^Ga-BZH3 indicated higher expression of bombesin receptors than that of the ανβ3 integrin in neuroendocrine tumors. ^68^Ga-BZH3 seems better suited for diagnosis of NETs owing to higher global tracer uptake.

## Introduction

During the past decade, the application of radiolabeled somatostatin analogs in nuclear medicine for diagnostics and therapy of neuroendocrine tumors has achieved success and stimulated the research in receptor targeting of additional tumor types [1]. Positron emission tomography (PET) is the most efficient imaging method in nuclear medicine because of its option of an absolute activity determination, its better contrast resolution, and its higher detection efficiency compared with conventional γ-cameras. PET with ^18^F-fluorodeoxyglucose (^18^F-FDG) is frequently used for oncologic applications to assess tissue viability, thereby gain the staging and therapy monitoring by qualitative analysis of SUV and quantitative evaluation based on the compartmental analysis of kinetic parameters [2]. However, not all tumors are ^18^F-FDG avid, and in particular treated tumorous lesions may demonstrate a low fluorodeoxyglucose (FDG) uptake and can therefore not be delineated using FDG. Therefore, new specific tracers are needed to enhance the sensitivity and specificity of PET. One approach is to study the expression of receptors to gain specificity. Experimental data demonstrated enhanced bombesin (BN) receptors in neuroendocrine tumors (NETs) [3-5].

Bombesin is an amphibian neuropeptide of 14 amino acids that shows a high affinity for the human gastrin-releasing peptide receptor (GRP-r, also known as BB_2_), which is overexpressed on several types of cancer. In addition, for the neuromedin B (BB_1_) and the bombesin receptor subtype (BB_3_), bombesin also shows a high affinity. Thus, radiolabeled BN and BN analogs may prove to be specific tracers for diagnostic and therapeutic targeting of GRP-r-positive tumors in nuclear medicine [6-13]. We have reported ^68^Ga-labeled bombesin may be helpful for diagnostic reasons in a subgroup of patients with GIST and recurrent gliomas [14,15].

The expression of GRP receptor in AR42J cell line has been reported by other groups [16,17]. So far, the expression of integrin ανβ3 in AR42J cell line has not been reported yet. However, the integrin ανβ3 plays an important role in angiogenesis and tumor metastasis. It is expressed on activated endothelial cells as well as some tumor cells [18]. Therefore, it is a promising imaging target as a potential surrogate parameter of angiogenic activity.

The ^68^Ga-RGD tetramer ^68^Ga-RGD_4 _is a specific tracer for the integrin ανβ3 [19]. Herein, dynamic PET studies with ^68^Ga-Bombesin were performed in AR42J tumor-bearing mice to investigate the impact of complementary receptor scintigraphy on diagnosis and the potential of a radionuclide treatment. Furthermore, dynamic ^68^Ga-RGD_4 _studies were performed for comparison.

## Materials and methods

### Synthesis of RGD_4_

Resins for peptide synthesis, coupling reagents, and Fmoc-protected amino acids were purchased from NovaBiochem. For analytical and semi-preparative high-performance liquid chromatography (HPLC), an Agilent 1200 system was used. The columns used for chromatography were a Chromolith Performance (RP-18e, 100 to 4.6 mm, Merck, Germany) and a Chromolith (RP-18e, 100-10 mm, Merck, Darmstadt, Germany) column, operated with flows of 4 and 8 mL/min, respectively. ESI and MALDI were obtained with a Finnigan MAT95Q and a Bruker Daltonics Microflex (Bruker Daltonics, Bremen, Germany), respectively.

The compound (DOTA-comprising maleimide tetramer (DOTA-Mal_4_)) was synthesized on solid support by standard Fmoc solid-phase peptide synthesis as described by Wellings et al. [20] on a standard rink amide resin. After coupling of Fmoc-Lys(Mtt)-OH to this resin (100 μmol), the Mtt-protecting group was removed by successive incubation with 1.75% TFA in DCM followed by coupling of tris-*t*Bu-DOTA and Fmoc-Lys(Fmoc)-OH under standard conditions. After removal of both lysine Fmoc protecting groups using deprotection times of twice 2 min and twice 5 min, Fmoc-Lys(Fmoc)-OH was coupled twice. After removal of all four lysine Fmoc protecting groups using deprotection times of twice 2 min and twice 10 min, maleimidobutyric acid was coupled applying the standard protocol. The product was cleaved from the solid support and deprotected using a mixture of TFA (trifluoroacetic acid)/TIS (triisopropylsilane)/H_2_O (95:2.5:2.5) for 45 min. The product was purified by semi-preparative HPLC using a gradient of 0% to 30% MeCN in 6 min and was obtained as a white solid upon lyophilization (49.8 mg, 31.6 μmol, 32%). ESI-MS (*m*/*z*) for [M + H]^+ ^(calculated): 1,576.76 (1,576.76) and [M + 2H]^2+ ^(calculated): 788.89 (788.88).

c(RGDfK)-PEG_1_-SH was synthesized on a preloaded Fmoc-Asp(NovaSyn TGA)-Oall resin (100 μmol) to which were subsequently coupled Fmoc-Gly-OH, Fmoc-Arg(Pbf)-OH, Fmoc-Lys(Mtt)-OH, and Fmoc-D-Phe-OH using standard coupling protocols. After allyl-deprotection, the peptide was cyclized and after removal of the Mtt-protecting group by successive incubation with 1.75% TFA in DCM, Fmoc-PEG_1_-OH, and SATA (*N*-succinimidyl-*S*-acetylthioacetate) were coupled. The product was cleaved from the solid support and deprotected using a mixture of TFA (trifluoroacetic acid)/TIS (triisopropylsilane)/H_2_O (95:2.5:2.5) for 45 min followed by an incubation with a hydroxylamine-containing solution (H_2_O + 0.1%TFA/MeCN + 0.1%TFA/50% hydroxylamine × HCl solution in water (750:750:25 μL)) for 5 min. The product was purified by semi-preparative HPLC using a gradient of 0% to 40% MeCN in 6 min and was obtained as a white solid upon lyophilization (33.2 mg, 40.4 μmol, 40%). ESI-MS (*m/z*) for [M + H]^+ ^(calculated): 823.38 (823.37).

The conjugation of c(RGDfK)-PEG_1_-SH to DOTA-Mal_4 _was carried out to yield DOTA-comprising RGD tetramer (DOTA-RGD_4_) as described before [21]. In brief, a solution of c(RGDfK)-PEG_1_-SH (15.6 mg, 19.0 μmol) in phosphate buffer (500 μL, 0.1 M, pH 6.0) was added to a solution of DOTA-Mal_4 _(5 mg, 3.2 μmol) in MeCN/phosphate buffer (0.1 M, pH 5.0) 1:1 (250 μL) and the pH of the mixture was adjusted to 7.4 by the addition of phosphate buffer (0.5 M, pH 7.4, approximately 100 μL). After 10 min, the product was purified by semi-preparative HPLC using a gradient of 0% to 40% MeCN in 6 min and was obtained as a white solid upon lyophilization (13.8 mg, 2.8 μmol, 89%). ESI-MS (*m*/*z*) for [M + K_complexed _+ 4H]^4+ ^(calculated): 1,227.05 (1,227.05) and (*m*/*z*) for [M + K_complexed _+ Na_salt _+ 4H]^4+ ^(calculated): 1,232.55 (1,232.55).

**Figure 1:**
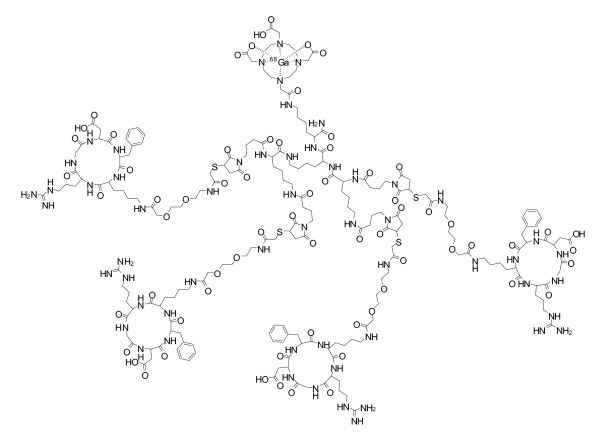
**The chemical structure of ^68^Ga-RGD_4_**.

DOTA is 1,4,7,10-tetraazacyclododecane-*N,N',N″,N'″*-tetraacetic acid. PEG is ethylene glycol (2-aminoethyl-carboxy-methyl ether). RGD is a cyclic pentapeptide containing the amino acid sequence D-Phe-Lys-Arg-Gly-Asp. Figure 1 shows the chemical structure of ^68^Ga-RGD_4_.

### Synthesis of BZH3

BZH3 was prepared according to the method described by Schuhmacher et al. [14]. BZH3 is DOTA-PEG_2_-[_D_-Tyr^6 ^-β-Ala^11 ^-Thi^13 ^-Nle^14 ^]BN(6-14) amide.

### Radiolabeling of BZH3 and RGD_4_

^68^Ga was used for labeling of both tracers and was obtained from a ^68^Ge/^68^Ga generator, which consists of a column containing a self-made phenolic ion-exchanger loaded with ^68^Ge and coupled in series with a small-sized anion-exchanger column (AG 1-X8 Cl^-^, mesh 200 to 400, Bio-Rad, Hercules, CA, USA) to concentrate ^68^Ga during elution. This generator provides ^68^Ga with an average yield of 60% for > 1.5 years. ^68^Ga-BZH3 and ^68^Ga-RGD_4 _were prepared according to the method described by Schuhmacher et al. and Jae Min Jeong et al., respectively [22,23]. The specific activity (the amount of radioactivity per peptide amount) of ^68^Ga-BZH3 and ^68^Ga-RGD_4 _were measured to be 28 and 22 MBq/nmol, respectively, which is sufficient for an efficient receptor imaging *in vivo*. Furthermore, a binding affinity of 4.973 μM (IC50) was obtained for ^68^Ga-RGD_4 _binding to ανβ3, which indicated that ^68^Ga-RGD_4 _could be used as PET tracer with ανβ3-positive neuroendocrine pancreatic tumor cell line.

### Cell lines

The AR42J cell line, derived from a rat exocrine pancreas neuroendocrine tumor, was used. Cells were obtained from the European Collection of Cell Cultures and were grown in RPMI 1640 medium supplemented with 2 mmol/L glutamine and 10% fetal calf serum. Adherent cells were dislodged with trypsin/ethylenediaminetetraacetic acid (0.02%: 0.05%, w/v). For PET-studies, 5 × 10^6 ^cells in 200 μl RPMI without supplements were inoculated subcutaneously in the right hind leg of Wistar rats.

### PET

**Figure 2:**
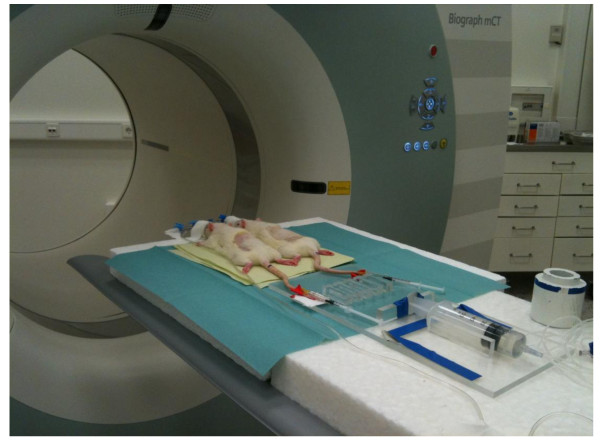
**Experimental setting**. Our experimental setting demonstrating two rats prior to positioning in the PET-CT scanner and homemade injector (two animals can be examined in parallel per scanning).

The study included nine AR 42 J tumor-bearing nude rats. We grouped all rats according to PET tracers (B, ^68^Ga-BZH3 and R, ^68^Ga-RGD_4_), used in dynamic PET scanning, as noted in Table 1. Dynamic PET studies were performed for 60 min after the intravenous application of 10 to 30 MBq ^68^Ga-RGD_4 _or 20 to 40 MBq ^68^Ga-BZH3, using a 28-frame protocol (ten frames of 30 s, five frames of 60 s, five frames of 120 s, and eight frames of 300 s). Two animals can be examined in parallel per scanning by a homemade injector (Figure 2). A dedicated PET-CT system (Biograph™ mCT, 128 S/X, Siemens Co, Erlangen, Germany) with an axial field of view of 21.6 cm with TrueV, operated in a three-dimensional mode, was used for all animal studies. The system provides the simultaneous acquisition of 369 transverse slices with a slice thickness of 0.6 mm. The animals were positioned in the axial plane of the system to maintain the best resolution in the center of the system. All PET images were attenuation-corrected and an image matrix of 400 × 400 pixels was used for iterative image reconstruction (voxel size 1.565 × 1.565 × 0.6 mm) based on the syngo MI PET/CT 2009C software version. After the end of the dynamic series an ultrahigh resolution CT with 85 mA, 80 kV and a pitch of 0.85 cm was performed for attenuation correction of the acquired dynamic emission data. The reconstructed images were converted to SUV images based on the formula [24]: SUV = Tissue concentration (becquerel per gram)/[injected dose (becquerel per gram))/body weight (gram)]. The SUV 55 to 60 min post-injection was used for the assessment of both tracers. The SUV images were used for all further quantitative evaluations.

**Table 1:** Quantitative PET parameters for the both tracers' kinetics of ^68^Ga-BZH3 and ^68^Ga-RGD_4_

Group		VB	*K*1	*k*2	*k*3	*k*4	RBP	FD	SUV	Number
	Mean	0.1037	0.3717	0.4808	0.1235	0.0986	0.0765	1.1198	1.0837	5
	SD	0.0443	0.0718	0.1312	0.0534	0.0524	0.0310	0.3800	0.1943	5
B	Median	0.0903	0.3506	0.5216	0.1177	0.1005	0.0607	1.1425	1.1865	5
	Minimum	0.0603	0.3055	0.2768	0.0580	0.0493	0.0506	1.0665	0.8628	5
	Maximum	0.1646	0.4862	0.6103	0.2005	0.1746	0.1202	1.1495	1.2884	5
	Mean	0.0582	0.2739	0.5386	0.1097	0.0443	0.0453	0.9961	1.0172	4
	SD	0.0102	0.0479	0.0985	0.0298	0.0052	0.0048	0.1184	0.2172	4
R	Median	0.0574	0.2728	0.5721	0.1180	0.0442	0.0442	1.0393	0.9886	4
	Minimum	0.0468	0.2304	0.3952	0.0678	0.0382	0.0414	0.8215	0.7833	4
	Maximum	0.0713	0.3195	0.6152	0.1352	0.0504	0.0514	1.0841	1.3084	4

^68^Ga-BZH3 (B, *n *= 5) and ^68^Ga-RGD_4 _(R, *n *= 4)

Dynamic PET data were evaluated using the software package PMOD (provided courtesy of PMOD Technologies Ltd., Zuerich, Switzerland) [25,26]. Areas with enhanced tracer uptake on transaxial, coronal, and sagittal images were evaluated visually. A volume of interest consists of several regions of interest over the target area. Irregular regions of interest were drawn manually. A detailed quantitative evaluation of tracer kinetics requires the use of compartmental modeling. A two-tissue-compartment model was used to evaluate the dynamic studies. This methodology is standard, particularly for the quantification of dynamic ^18^F-FDG studies [27,28].

In animals, a partial volume correction must be applied to the data due to the small size of the input and tumor volumes of interest (VOIs). Herein, the recovery coefficient was 0.85 for a diameter of 8 mm and 0.32 for a diameter of 3 mm based on phantom measurements as well as the recent parameter settings used with the reconstruction software. For the input function the mean values of the VOI data obtained from the heart were used. We used a preprocessing tool, which allowed a fit of the input curve by a sum of up to three decaying exponentials. The learning-machine two-tissue-compartment model was used for the fitting and provided five parameters: the transport parameters for tracer into and out of the cell, *K*1 and *k*2, the parameters for phosphorylation and dephosphorylation of intracellular tracer, *k*3 and *k*4, and the fractional blood volume, also called vessel density (VB), which reflects the amount of blood in the VOI. Following compartment analysis, we calculated the global influx of tracer from the compartment data using the formula: influx = (*K*1 × *k*3)/(*k*2 + *k*3). Compared to the standard iterative method, the machine learning method has the advantage of a fast convergence and avoidance of over fitting [29]. The model parameters were accepted when *K*1 - *k*4 was less than 1 and VB exceeded 0. The unit for the rate constants *K*1 to *k*4 was 1/min. In the case of ^68^Ga-BZH3 and ^68^Ga-RGD_4_, *K*1 is associated with receptor binding, *k*2 with displacement from the receptor, *k*3 with cellular internalisation, and *k*4 with externalisation.

Besides the compartmental analysis, a non-compartmental model based on the fractal dimension was used. The fractal dimension is a parameter of heterogeneity and was calculated for the time-activity data of each individual volume of interest. The values fro fractal dimension vary from 0 to 2, showing the deterministic or chaotic distribution of tracer activity. We used a subdivision of 7 × 7 and a maximal SUV of 20 for the calculation of fractal dimension [30].

### Statistical analysis

Statistical evaluation was performed with Stata/SE 10.1 (StataCorp, College Station, TX, USA). Statistical evaluation was performed using the descriptive statistics and scatter plots. The classification analysis was performed using the GenePET software [31]. The software applies the support vector machines (SVM) algorithm and provides a classification analysis by optimizing a hyperplane between the target variables. The algorithm for selection or elimination of variables, the feature ranking, can be based on different criteria, e.g., *F *test, Mann-Whitney test, or the SVM ranking feature elimination (SVM RFE) approach [32]. The SVM RFE algorithm computes a multidimensional weight vector for the PET variables and the square of the vector is used to calculate the ranking criteria. For comparison between two tracers, the two-sided Wilcoxon rank-sum test was applied for all PET parameters, SUV, and the fractal dimension (FD), using a single parameter analysis. *P *values < 0.05 were considered significant.

## Results

**Figure 3:**
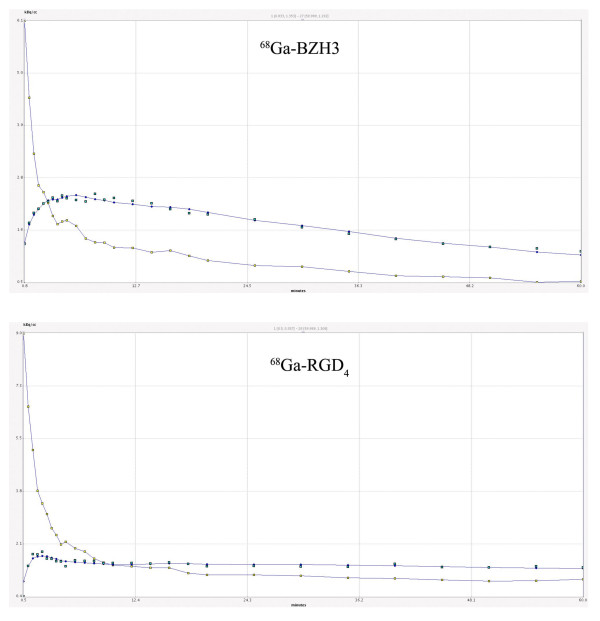
**Representative blood input function (filled yellow squares) and tumor tissue tracer concentrations**. Obtained in a 60-min acquisition sequence (left, ^68^Ga-BZH3; right, ^68^Ga-RGD_4_). Filled green squares are tracer concentrations for each image, while the smooth line through the data is the two-tissue-compartment model fit to the data. A smooth line (interpolation) was drawn through the input function data to illustrate the shape of the curve.

Figure 3 is a representative set of time-activity data obtained with a image-derived measured blood input function, which illustrates the good statistical quality of the data and model fit using nonlinear regression and two-tissue-compartment model.

Table 1 presents the mean, median, minimum, and maximum values as well as the standard deviation for the SUV, FD, and kinetic values of all parameters for both tracers (Table 1). In the whole paper, B and R represent ^68^Ga-BZH3 and ^68^Ga-RGD_4 _respectively. The Wilcoxon rank-sum test was used to reveal statistically significant differences between all variables.

**Figure 4:**
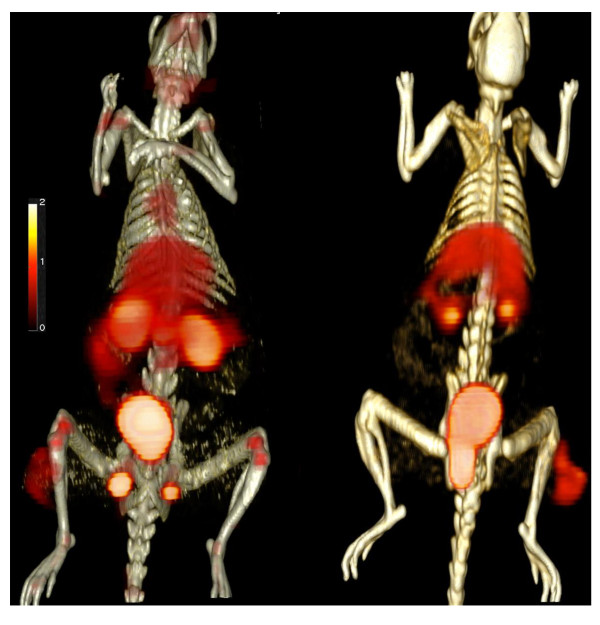
**3D fused PET-CT images for ^68^Ga-RGD_4 _(left) and ^68^Ga-Bombesin (right)**. Besides the tumor region in the lower leg. The tracer uptake in other sites covered heart (exhibit for ^68^Ga-RGD_4_), liver, kidney, urinary bladder, and testicles (left for male rat).

Figure 4 shows an example of 3D fused PET-CT images for ^68^Ga-RGD_4 _and ^68^Ga-Bombesin. The ^68^Ga-BZH3 image clearly showed enhanced ^68^Ga-BZH3 uptake in the tumor area in the lower leg. ^68^Ga-BZH3 uptake in the evaluated tumor lesions was generally higher than ^68^Ga-RGD_4 _uptake.

**Figure 5:**
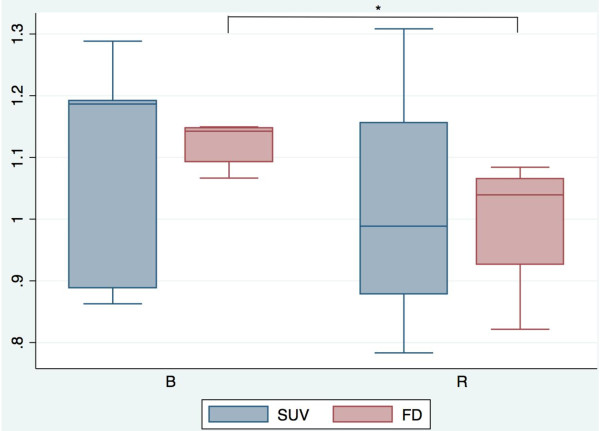
**Box-Whiskers plots of the median SUV 55 to 60 min and FD for both tracers**. *P values < 0.05. B, ^68^Ga-BZH3; R, ^68^Ga-RGD_4_.

**Figure 6:**
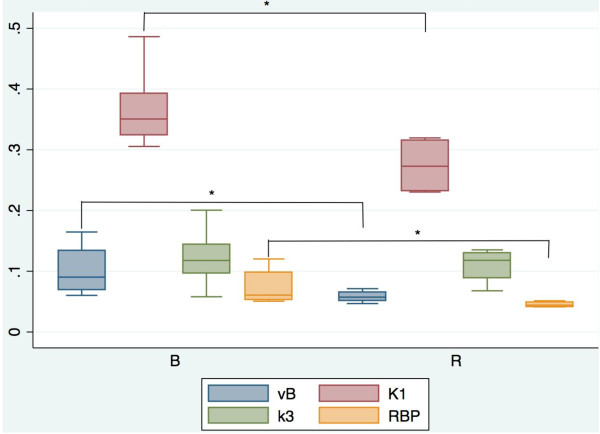
**Box-Whiskers plots of median values for both tracers for vB, *K*1, *k*3 and RBP**. *P values < 0.05). B, ^68^Ga-BZH3; R, ^68^Ga-RGD_4_.

Box plots of ^68^Ga-BZH3 and ^68^Ga-RGD_4 _uptake (56- to 60-min SUV) in tumor tissue and FD are presented in Figure 5. The corresponding quantitative data and the corresponding *P *values are presented in Table 1 and 2. The 56- to 60-min SUV for ^68^Ga-BZH3, with a range of 0.86 to 1.29 (median 1.19) was higher than the corresponding value for ^68^Ga-RGD_4_, with a range of 0.78 to 1.31 (median 0.99). However, there was no significant difference in the median SUV between the two tracers. Interestingly, the median FD for ^68^Ga-BZH3 (1.1425) was significantly higher as compared with ^68^Ga-RGD_4 _(1.039) (*P *= 0.0275) at a level of *P *< 0.05. The transport rate constants *K*1 and *k*3 (1/min), the receptor binding potential (RBP) and the vascular fraction VB of both tracers are presented in Figure 6. Kinetic data demonstrate higher the values of VB for ^68^Ga-BZH3 as compared with ^68^Ga-RGD_4 _(0.0903 vs. 0.0574); furthermore, VB was relatively low for both tracers, not exceeding 0.2. Furthermore, the values of *K*1 and RBP were higher for ^68^Ga-BZH3 than the corresponding values for ^68^Ga-RGD_4 _(0.3506 vs. 0.2728 and 0.0607 vs. 0.0442, respectively). In addition, comparable *k*3 values without significant difference for both tracers were displayed in Figure 6.

**Table 2:** The value of statistically significant level *P *using the Wilcoxon rank-sum test

*P*							
**VB**	***K*1**	***k*2**	***k*3**	***k*4**	**RBP**	**SUV**	**FD**

0.05*	0.05*	0.4624	0.8065	0.05*	0.0275*	0.8065	0.0275*

*P values < 0.05 were considered significant.

## Discussion

PET with FDG is frequently used for oncological application to assess tissue viability. However, owing to the low FDG uptake in some tumor types, like in the neuroendocrine carcinomas, there is a need for new radiotracers. One idea is to study the expression of different receptors in order to guide diagnostics and even more therapy in that direction, e.g. using a radionuclide-based therapy. NETs originate mostly from the gastroenteropancreatic tract and express specific receptors like amine and peptide receptors (somatostatin, vasointestinal peptide receptors, bombesin, cholecystokinin, gastrin and/or substance P) [33]. Adams et al. reported the comparison of different tracers in detecting malignant NETs and revealed that increased FDG uptake was associated with malignancy [34]. In nude mice bearing the AR4-2J tumor, tumor uptake of both ^90^Y and ^111^In-DOTATOC 4 h after injection was five times higher than with ^111^In-DTPA-octreotide [35]. We had reported on ^68^Ga-DOTATOC studies in patients with NETs and an enhanced uptake in metastases of NETs [36]. Furthermore, we have shown that the global DOTATOC uptake in NETs is mainly dependent on *k*1 (receptor binding) and VB (fractional blood volume) and less on the *k*3 (internalization). ^68^Ga-DOTATOC was better suited than ^18^F-FDG for the diagnosis of metastatic NETs. The ^68^Ga-DOTATOC uptake was also used as a parameter for a radionuclide therapy with ^90^Y-DOTATOC. Patients with lesions demonstrating an enhanced ^68^Ga-DOTATOC uptake (> 5.0 SUV) were selected for radionuclide therapy [37].

Bombesin and the two mammalian bombesin-like peptides, BB1 and BB2 regulate many biologic response processes through activation of distinct receptor subtypes, including modulation of smooth muscle contraction, secretion of neuropeptides and hormones, as well as stimulation of cell growth [38,39]. Activation of neuromedin B (BB1) receptors has been reported in various human cancers [39]. Experimental studies demonstrated an enhanced bombesin receptor expression in several human adult glioblastoma cell lines as well as in two pediatric human glioblastoma cell lines [38]. We reported on an enhanced ^68^Ga-BZH3 uptake in a subgroup of patients with gastrointestinal stromal tumors [15], and quantitative ^68^Ga-BZH3 studies were helpful in patients with recurrent gliomas for tumor grading and the differentiation between high- and low-grade tumors [14]. In addition, other bombesin analogues ^64^Cu-, ^99m^Tc-, ^188^Re-, ^177^Lu-, ^90^Y-, and ^111^In have been reported to be promising radiotracers for PET imaging of many human cancers overexpressing the GRP receptor such as breast cancer and prostate carcinoma [6-13,40,41].

Integrins play a key role in angiogenesis and tumor metastasis by mediating tumor cell invasion and movement across blood vessel, whereas integrins expressed on endothelial cells modulate cell migration and survival during the angiogenic cascade. A common feature of many integrins like ανβ3 is that they bind to extracellular matrix proteins via the three amino acid sequence arginine-glycine-aspartic acid (RGD) [42,43]. Radiolabeled RGD-peptides, the integrin ανβ3-specific tracers, have been developed for PET and SPECT imaging. A mass of data suggested that ανβ3 expression can be quantified by radiolabeled RGD-peptides [44-46]. In this study, ^68^Ga-BZH3 and ^68^Ga-RGD_4 _were used as tracers for PET to assess the receptor expression in AR42J tumor-bearing nude rats by comparison.

Quantitative dynamic PET provides the possibility for absolute tracer quantification and is superior to static images, which are widely used, but do not provide information on tracer kinetics. Furthermore, the use of a two-compartment model is the superior approach for the assessment of tracer kinetics, and is accepted for research purposes [27]. Concerning the ^68^Ga-BZH3 kinetics, *k*1 is a parameter that reflects the receptor binding and *k*3 is a parameter that reflects the internalization of the tracer. A lower receptor binding of ^68^Ga-BZH3 was reported in gliomas as compared with ^68^Ga-DOTATOC in meningiomas, but higher internalization, were proved [47]. In the present study, the comparison of the ^68^Ga-BZH3 kinetics with the ^68^Ga-RGD_4 _kinetics in the ARJ 42 tumor-bearing nude rats revealed higher mean values of *k*1 for ^68^Ga-BZH3 (median, 0.3506) as compared with ^68^Ga-RGD_4 _(median 0.2728), and comparable *k*3 values (median, 0.1177 vs. 0.1180). According to these data, the tracers' accumulation in this neuroendocrine tumor cell line is primarily depends on the receptor binding and less on the internalization.

Generally, ^68^Ga-BZH3 uptake was lower than ^18^F-FDG [15]. Herein, we found ^68^Ga-BZH3 uptake was higher than that of ^68^Ga-RGD_4_, and the values were relatively comparable in comparison to that reported in gliomas [14]. In particular, there were significant differences between VB, *K*1, *k*4, RBP, and FD. The fractional blood values VB of ^68^Ga-BZH3 were higher than that of ^68^Ga-RGD_4 _(median, 0.0903 vs. 0.0574), however for both tracers they are low in comparison to those reported for other tracers, like ^68^Ga-DOTATOC and ^18^F-FDG. This is in accordance to previous published data, e.g. in melanoma patients and confirm the hypothesis that the absolute value of VB depend on the applied tracer [48]. The VB and RBP values for ^68^Ga-BZH3 were more spread out than those determined for ^68^Ga-RGD_4_. A possible explanation is that the tracer uptake of ^68^Ga-RGD_4 _was generally lower than that of ^68^Ga-BZH3.

Cancer is often characterized by chaotic, poorly regulated growth. Recent studies have shown that fractal geometry can be useful to describe the pathological architecture of tumors and angiogenesis. Fractals can be useful measures of pathologies of the vascular architecture, the tumor border, and the cellular morphology [49]. The FD is used to characterize the chaotic nature of the tracer's distribution in primary tumors and metastases, based on the box counting procedure of chaos theory, for the analysis of dynamic PET data. In the present study, FD values for ^68^Ga-BZH3 were ranged from 1.066 to 1.150 (median, 1.142), higher than that for ^68^Ga-RGD_4 _(median, 0.989), but both are lower compared with those measured in malignancies with different tracers, such as ^68^Ga-DOTATOC, ^18^F-FDG, ^15^O-water, and ^18^F-DOPA (a median FD exceed 1.25) [48,50].

## Conclusion

In general, a high SUV indicates high receptor binding. The preliminary results give evidence for a higher BZH3 uptake, which is related to higher bombesin and neuromedin B gene expression than that of ανβ3 in neuroendocrine tumors. ^68^Ga-BZH3 seems better for diagnosis of NETs owing to higher values of global tracer uptake. Further studies with a larger number of animals and in patients are needed to confirm these preliminary results.

## Competing interests

The authors declare that they have no competing interests.

## Authors' contributions

CC participated in the whole study and wrote the manuscript. LP performed the statistical analysis. MS, CW, and BW carried out the synthesis of tracers. ADS and LGS did the design of the study and revised the whole manuscript. UH gave financial support. All authors read and approved the final manuscript.
